# Free Cortisol and Free 21-Deoxycortisol in the Clinical Evaluation of Congenital Adrenal Hyperplasia

**DOI:** 10.1210/clinem/dgae591

**Published:** 2024-08-26

**Authors:** Bas P H Adriaansen, Agustini Utari, André J Olthaar, Rob C B M van der Steen, Karijn J Pijnenburg-Kleizen, Lizanne Berkenbosch, Paul N Span, Fred C G J Sweep, Hedi L Claahsen-van der Grinten, Antonius E van Herwaarden

**Affiliations:** Department of Laboratory Medicine, Radboud University Medical Center, 6525 GA Nijmegen, the Netherlands; Department of Pediatrics, Division of Pediatric Endocrinology, Amalia Children's Hospital, Radboud University Medical Center, 6525 GA Nijmegen, the Netherlands; Department of Pediatrics, Division of Pediatric Endocrinology, Faculty of Medicine, Diponegoro University, Jawa Tengah 50275, Semarang, Indonesia; Department of Laboratory Medicine, Radboud University Medical Center, 6525 GA Nijmegen, the Netherlands; Department of Laboratory Medicine, Radboud University Medical Center, 6525 GA Nijmegen, the Netherlands; Department of Pediatrics, Canisius Wilhelmina Hospital, 6532 SZ Nijmegen, the Netherlands; Department of Pediatrics, Division of Pediatric Endocrinology, Amalia Children's Hospital, Radboud University Medical Center, 6525 GA Nijmegen, the Netherlands; Department of Pediatrics, Division of Pediatric Endocrinology, MosaKids Children's Hospital, Maastricht University Medical Centre+, 6229 HX Maastricht, the Netherlands; Department of Radiation Oncology, Radboud University Medical Center, 6525 GA Nijmegen, the Netherlands; Department of Laboratory Medicine, Radboud University Medical Center, 6525 GA Nijmegen, the Netherlands; Department of Pediatrics, Division of Pediatric Endocrinology, Amalia Children's Hospital, Radboud University Medical Center, 6525 GA Nijmegen, the Netherlands; Department of Laboratory Medicine, Radboud University Medical Center, 6525 GA Nijmegen, the Netherlands

**Keywords:** ACTH, CAH, cortisol, free cortisol, 21-deoxycortisol, free 21-deoxycortisol

## Abstract

**Context:**

Some patients with classic congenital adrenal hyperplasia (CAH) survive without glucocorticoid treatment. Increased precursor concentrations in these patients might lead to higher free (biological active) cortisol concentrations by influencing cortisol–protein binding. In 21-hydroxylase deficiency (21OHD), the most common CAH form, accumulated 21-deoxycortisol (21DF) may further increase glucocorticoid activity. Both mechanisms could explain the low occurrence of symptoms in some patients with untreated classic CAH.

**Objective:**

Develop and validate a liquid chromatography tandem mass spectrometry (LC-MS/MS) method for free cortisol and free 21DF to quantify these steroids in patients with untreated classic CAH before and after Synacthen administration, and compare these concentrations to concentrations measured in patients with nonclassic CAH (NCCAH), other forms of adrenal insufficiency (AI), and controls.

**Methods:**

An LC-MS/MS method to measure free cortisol and free 21DF was developed and validated. Total and free serum concentrations of both cortisol and 21DF were measured in patients with untreated classic CAH (n = 29), NCCAH (n = 5), AI (n = 3), and controls (n = 11) before and 60 minutes after stimulation with Synacthen.

**Results:**

Unstimulated total cortisol concentrations of patients with untreated classic CAH (median 109 nmol/L) were lower than in patients with untreated NCCAH (249 nmol/L, *P* = .010) and controls (202 nmol/L, *P* = .016), but free cortisol concentrations were similar. Basal free 21DF concentrations were high in patients with 21OHD (median 5.32 nmol/L) and undetectable in patients with AI and controls (<0.19 nmol/L). After Synacthen administration, free 21DF concentrations increased in patients with 21OHD, while free cortisol concentrations did not change.

**Conclusion:**

Free cortisol concentrations in patients with classic CAH were similar to those in controls and patients with NCCAH, indicating comparable cortisol availability. Additionally, patients with 21OHD produce high concentrations of 21DF, possibly explaining the low occurrence of symptoms in some patients with classic 21OHD. Free cortisol and 21DF levels should be considered in evaluating adrenal insufficiency in patients with CAH.

Glucocorticoids, such as cortisol, are crucial for regulating blood pressure, glucose metabolism, and the immune response (1, 2). Cortisol is produced by the adrenal cortex and has a diurnal rhythm with the highest concentration in the early morning (3). This rhythm is regulated by the hypothalamus through stimulation of the pituitary gland, which releases adrenocorticotropic hormone (ACTH). Negative feedback by cortisol balances the hypothalamus–pituitary–adrenal axis (3). During stress, such as infection or surgery, the cortisol demand increases, which is mediated by an increased ACTH release (4).

Primary adrenal insufficiency (PAI) results in inadequate cortisol production and an inability to increase cortisol concentrations during stress, leading to symptoms like extreme fatigue, weight loss, and abdominal pain (5). In adults, PAI is most often caused by acquired conditions such as autoimmune adrenalitis (Addison disease). In children, inherited conditions are most common (6), such as classic congenital adrenal hyperplasia (CAH), primarily due to 21-hydroxylase deficiency (21OHD) (90-95%) or 11-hydroxylase deficiency (11OHD) (∼5%) (7). Patients with these conditions produce insufficient cortisol concentrations with consequently reduced negative feedback to the pituitary gland, causing chronically elevated ACTH levels. This leads to continuous activation of the adrenal cortex and accumulation of precursor steroids before the enzymatic block (7), which are partly shunted into the unaffected adrenal androgen pathway, leading to hyperandrogenism (8, 9). In contrast to other forms of PAI, the hallmark of classic CAH is not only a decreased concentration of cortisol but also strongly elevated concentrations of precursor steroids and adrenal androgens. Patients with nonclassic CAH (NCCAH), a less severe subgroup, generally produce normal cortisol concentrations but still have elevated concentrations of precursor steroids and androgens (4).

Diagnosis of PAI involves measuring morning total cortisol concentrations in blood. To assess hypothalamus–pituitary–adrenal axis responsiveness, stimulation tests, such as the insulin tolerance test (10) or Synacthen test (11), are used. In these tests, a suboptimal cortisol response is defined as a total cortisol concentration <500 nmol/L (18 μg/dL) in the PAI guideline (5), but there is a debate on this threshold (12). The CAH guideline specifies a cutoff of <400 to 500 nmol/L (14-18 μg/dL), depending on the measuring method (13).

Cortisol in blood is mostly bound to proteins (±95%), such as corticosteroid-binding globulin (CBG) and albumin (14, 15). The measured total cortisol comprises the bound and free (ie, unbound) cortisol, while only the latter determines the biological glucocorticoid activity according to the free hormone hypothesis (16). In patients with 21OHD, elevated levels of 17-hydroxyprogesterone (17OHP) can be metabolized by 11-hydroxylase into 21-deoxycortisol (21DF) (7). Interestingly, previous studies have described untreated patients with classic CAH with insufficient cortisol production without overt complaints of cortisol deficiency (17-20). Our group has demonstrated that 21DF can transactivate the glucocorticoid receptor with 49% of cortisol's potency (17). Hence, 21DF contributes to glucocorticoid activity, particularly at elevated concentrations as observed in patients with 21OHD (17). Furthermore, precursor steroids can bind to CBG (15, 21, 22), thereby competing with cortisol for bindings spots and preventing cortisol from binding, therewith increasing the free fraction of cortisol. These mechanisms might contribute to the attenuation of symptoms in patients with untreated classic CAH.

So far, no studies have been conducted on free cortisol and free 21DF measurements in patients with CAH. Our aim is to quantify total and free concentrations of cortisol and 21DF in the morning and 60 minutes after administration of Synacthen in patients with untreated classic CAH (both 11OHD and 21OHD). We compare these results with patients with NCCAH, patients with other forms of adrenal insufficiency (AI), and controls to investigate the situation of patients with CAH in more detail. We hypothesize that free cortisol and (free) 21DF concentrations might give a better reflection of the glucocorticoid activity in patients with 21OHD than solely measuring total cortisol concentrations.

## Materials and Methods

### Subjects

This study was approved by the local medical ethics committee (Radboudumc, case number 2021-12944). Patients were included via 3 routes (see Fig. S1 (23)):

1. Indonesian cohort (n = 22): Serum samples from untreated patients with classic CAH (21OHD, n = 17; 11OHD, n = 5) were obtained from an Indonesian cohort as previously described (17). Ethical approval was obtained from the ethics committee of the Faculty of Medicine, Diponegoro University, Semarang, Indonesia (713/EC/FK-RSDK/2016).
2. Dutch retrospective cohort (n = 39): Stored residual serum samples from Synacthen tests performed between January 2016 and March 2021 at the Radboud university medical center were used.
3. Dutch prospective cohort (n = 19): Residual serum and plasma samples, both Synacthen tests and random samples, were obtained from 3 Dutch hospitals between April 2021 and January 2024.

For the Synacthen tests, blood was obtained before (T0) and 60 minutes after (T60) administration of 250 μg of tetracosactide. Samples were kept frozen at ≤−30 °C until analysis. Inclusion criteria were ≥300 µL of residual serum available and patients received no systemic glucocorticoid treatment for at least 1 year. Pseudonymized data about age, gender, time of blood withdrawal, indication of Synacthen test, and diagnosis were collected. All patients were diagnosed following the current guidelines (5, 13, 24). They were divided into 4 groups: (1) patients with classic CAH (both 21OHD and 11OHD), (2) patients with NCCAH based on 21OHD, (3) patients with other forms of AI, and (4) patients with an optimal cortisol response after Synacthen (controls). Genotyping was performed on all patients diagnosed with biochemically confirmed adrenal insufficiency to identify possible genetic causes. For the patients with AI, CAH was ruled out by clinical evaluation, biochemistry, and genotyping. Classification of patients with CAH into classic CAH and NCCAH was based on clinical characteristics, biochemical evaluation, and genetic analysis (Table S1 (23)) (25, 26). Sequencing results were compared with the reference sequence GenBank NM_000500.9 for *CYP21A2* and NM_000497.3 for *CYP11B1*. In this article the term “classic CAH” refers to both patients with 21OHD and patients with 11OHD. All patients with classic CAH and with AI were counseled about glucocorticoid treatment, but some Indonesian patients declined treatment due to the lack of complaints. All Dutch patients with classic CAH and patients with AI were treated with glucocorticoid treatment according to the current guidelines (5, 13). Blood samples analyzed in this study were taken before initiation of glucocorticoid treatment.

### Measurement of Total Steroid Concentrations With Liquid Chromatography Tandem Mass Spectrometry

Total serum 17OHP (17-hydroxypregn-4-ene-3,20-dione), 11-deoxycortisol (11DF; 17,21-dihydroxypregn-4-ene-3,20-dione), 21DF (11,17-dihydroxypregn-4-ene-3,20-dione), and cortisol (11,17,21-trihydroxypregn-4-ene-3,20-dione) were analyzed by a clinically validated liquid chromatography tandem mass spectrometry (LC-MS/MS) method on a 6490 triple quadrupole LC-MS/MS (Agilent Technologies, Santa Clara, CA) preceded by protein precipitation and solid phase extraction (SPE). An internal standard mix (20 µL in methanol:H_2_O 30:70) containing [^13^C_3_]-17OHP, [^2^H_5_]-11-deoxycortisol, [^2^H_4_]-21DF, and [^13^C_3_]-cortisol (all from Isosciences, Ambler, PA) with concentrations of 10, 10, 20, and 100 nmol/L, respectively, was added to 100 µL of serum. For protein precipitation, acetonitrile + 0.1% formic acid was added and incubated for at least 10 minutes at room temperature and centrifuged (10 minutes, 5000*g*). Then, 200 μL of supernatant was added to 300 μL of H_2_O followed by SPE using Oasis HLB 1-cc cartridges (Waters Corporation, Milford, MA). These cartridges were preequilibrated with 1 mL of methanol:isopropanol (95:5) and consequently washed with 1 mL of H_2_O. After sample application, columns were washed with 1 mL of H_2_O and 1 mL of methanol:H_2_O (30:70). The eluate (300 μL, methanol:isopropanol 95:5) was dried under a stream of N_2_ gas at 40 °C, reconstituted in 100 μL of methanol:H_2_O (30:70), and injected (10 µL) into an Agilent Technologies 1290 Infinity UHPLC-system (Agilent Technologies). LC-MS/MS settings are shown elsewhere (Table S2 (23)). The collision energy was optimized for each analyte and internal standard and 2 mass transitions (quantitative and qualitative) were monitored. A 9-point calibration curve with 17OHP, 11DF, 21DF, and cortisol (all from Sigma-Aldrich, St. Louis, MO) was used for quantification. The calibration ranges were 0 to 167 nmol/L for 17OHP, 0 to 191 nmol/L for 11DF, 0 to 167 nmol/L for 21DF, and 0 to 2299 nmol/L for cortisol. In-house quality controls of human serum were measured in duplicate in each run.

### Measurement of Free Steroid Concentrations With LC-MS/MS

Measurement of free 21DF was incorporated in a clinically validated measuring method for free cortisol and free testosterone. Equilibrium dialysis was performed with samples buffered to pH 7.4 (±0.03) at 37 °C (±0.5) by a previously described HEPES buffer (4-(2-Hydroxyethyl)piperazine-1-ethanesulfonic acid; Merck, Rahway, NJ) that resembled the ionic environment of serum (27). Two identical volume compartments (180 μL) were separated by a semipermeable regenerated cellulose membrane (Serva Electrophoresis GmbH, Heidelberg, Germany) with a diameter of 28 mm and maximum permeability of 5 kDA. During dialysis, cells were placed in a water bath at 37 °C (±0.5) for 5 hours while continuously rotating. After dialysis, dialysate was emptied from the cell and SPE was performed with 100 μL preceded by the addition of H_2_O (30 μL) and an internal standard mix (20 μL) containing [^2^H_4_]-21DF, [^13^C_3_]-cortisol, and [^13^C_3_]-testosterone (all from IsoSciences). For SPE, Oasis MCX 1-cc cartridges (Waters Corporation) were preequilibrated with 1 mL of methanol:isopropanol (95:5) and consequently washed with 1 mL of H_2_O. After sample application, columns were washed with 1 mL of H_2_O + 5% ammonium hydroxide and 1 mL of methanol:H_2_O (20:80) + 2% formic acid. The eluate (300 μL, 100% methanol) was dried in the Savant SpeedVac Concentrator (ThermoFisher Scientific, Waltham, MA) for ±1 hour, reconstituted in 30 μL of methanol:H_2_O (30:70) and injected (10 µL) into a Xavi TQ-XS StepWave XS triple quadrupole spectrometer (Waters Incorporation). LC-MS/MS settings including collision energy and mass transitions are shown elsewhere (Table S3 (23)). A 9-point calibration curve with 21DF and cortisol (both from Sigma-Aldrich) was used for quantification. The calibration ranges were 0 to 115 nmol/L for both steroids.

### Method Validation

All measurements were performed in an ISO15189 accredited laboratory. All clinically validated measuring methods are monitored for quality in each run using in duplicate measurements of in-house quality controls of human serum. For validation, a CLSI EP6 protocol (28) in serum was used to assess linearity. We measured steroid concentrations in a blank sample that was injected after the highest standard to assess carryover. Ion suppression was measured according to CLSI C62-A guideline (29): qualitative ion suppression by continuous infusion of the labeled steroid, and quantitative ion suppression by comparing the signal of human serum measurements (n = 5) with the signal of spiked prepared matrix-free blank. Imprecision was assessed by a modified CLSI EP5 protocol (30) with pooled human serum samples at a low (n = 7 for 21DF; n = 20 for cortisol) and high concentration (n = 8 for 21DF, n = 20 for cortisol). The lower limit of quantification (LLOQ) was defined as the lowest concentration with an interassay coefficient of variation <20% and was determined with a modified evaluation protocol from pooled serum by between-day assay repeated measurements (n = 10). For 21DF, recovery was calculated in 5 samples to which 29.94 nmol/L was added. For cortisol, a National Institute of Standards and Technology standard (LOT number: 271020) diluted to 1.496 nmol/L was measured in each run (n = 29). A correction factor of 1.03 was applied to adjust free cortisol levels according to the National Institute of Standards and Technology standard. Possible interfering substances were identified by measuring commercially available steroids and antiandrogens with a molecular mass ≤2 g/mol lower than the analyte or the internal standard. First, retention time interference was checked, and, second, the multiple reaction monitoring transition interference was evaluated.

### Data and Statistical Analyses

Free cortisol percentage was calculated by dividing the free cortisol concentration by the total cortisol concentration from the same sample multiplied by 100%. Since cortisol has a diurnal rhythm, only morning samples (between 08:00 and 11:00 Am) were used for the analyses of absolute free and total cortisol concentrations. Results less than the LLOQ with a signal to noise ratio <5 were assigned a value of half the LLOQ. These results were excluded from the calculation of percentages. A Kruskal–Wallis test with post hoc Dunn's test was used to compare means between different subgroups (classic CAH vs NCCAH vs control). For comparison of related samples (T0 vs T60), a related-samples Wilcoxon signed rank test was used. Patients from the AI group were excluded for statistical comparison due to the low number of inclusions.

## Results

### Subjects

Data from 22 Indonesian and 58 Dutch patients were collected (see Fig. S1 (23) for the inclusion flowchart). Patients with insufficient residual material (n = 31) and those treated with glucocorticoids before the test (n = 1) were excluded. In total, 48 patients were divided into 4 groups: classic CAH (n = 29; 21OHD [n = 24] and 11OHD [n = 5]), NCCAH (n = 5), AI (n = 3), and controls (n = 11). Patient demographics are shown in Table 1.

**Table 1.** Demographics of included patients

	CAH	NCCAH	AI	Control
Number (n)	29	5	3	11
Age (years)	13.2 (3.3-19.6)	8.9 (6.0-18.7)	16.1 (11.8-17.3)	9.8 (7.9-14.7)
Genotype (% 46,XY)	10.3	40.0	33.3	63.6
Sex (% males)	41.4	40.0	33.3	63.6
Diagnosis/Indication for Synacthen test	CAH (100%)	NCCAH (100%)	X-ALD (33.3%) AD (33.3%) Unknown (33.3%)	Screening X-ALD (18%) Premature pubarche (18%) Screening hypothyroidism (18%) Fatigue (9%) Collapse and hypotension (9%) Screening MEN-1 (9%) Rapid postnatal growth (9%) Once measured low cortisol (9%)

Abbreviations: AD, Addison disease; AI, adrenal insufficiency; CAH, congenital adrenal hyperplasia; MEN-1, multiple endocrine neoplasia 1; NCCAH, nonclassic CAH; X-ALD, X-linked adrenoleukodystrophy.

Continuous data are given as median (interquartile range).

### Method Validation

An LC-MS/MS method for measuring free cortisol and free 21DF was established and validated. The method was linear between 0.13 and 99.2 nmol/L for 21DF and between 0.08 and 66 nmol/L for cortisol. Table 2 shows the maximum deviation from linearity, imprecision, LLOQ, recovery, and ion suppression. Carryover was negligible for both components. For cortisol and its internal standard, we evaluated 18-hydroxycorticosterone as a possible interfering substance, and it showed no interference. For 21DF and its internal standard, corticosterone, 11-deoxycortisol, and 18-hydroxy-11-deoxycorticosterone were evaluated and showed no interference.

**Table 2.** Linearity, imprecision (within and between day) on low and high level, LLOQ, recovery, and ion suppression for free 21DF and free cortisol measured by LC-MS/MS after dialysis

	Free 21DF	Free cortisol
**Linearity**		
Linearity range (nmol/L)	0.13-99.2	0.08-66.0
Deviation from linearity (%)	1.55	0.96
**Imprecision low**		
Concentration (nmol/L)	0.36	11.4
Within day (%)	2.8	2.6
Between day (%)	8.5	4.6
**Imprecision high**		
Concentration (nmol/L)	4.89	104.4
Within day (%)	3.3	2.5
Between day (%)	2.8	4.0
LLOQ (nmol/L)	0.19	<0.32*^a^*
**Recovery**		
Concentration (nmol/L)	29.94	1.496
Average recovery (%)	108	97.3
Ion suppression (%)	<1	<1

Abbreviations: 21DF, 21-deoxycortisol; LC-MS/MS, liquid chromatography tandem mass spectrometry; LLOQ, lower limit of quantification.

^*a*^Mean coefficient of variation of 6.1%.

### Patients with Classic CAH and Controls Have Similar Free Morning Cortisol Concentrations

**Figure 1.**
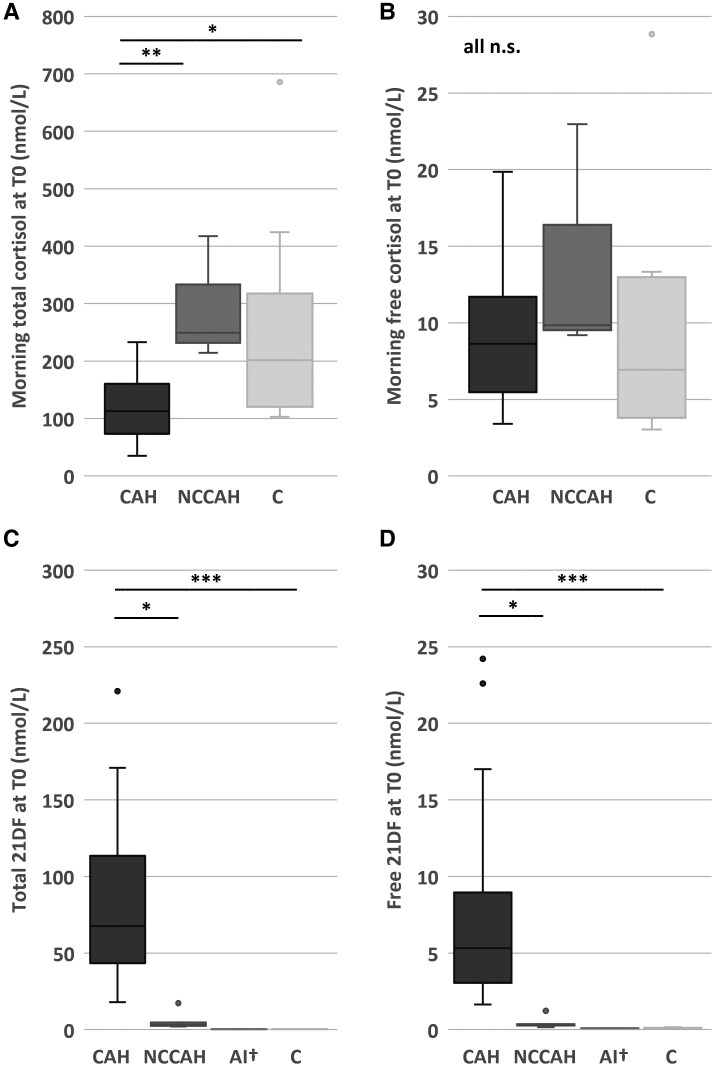
Steroid concentration (nmol/L) at T0 in patients with classic congenital adrenal hyperplasia (CAH), nonclassic congenital adrenal hyperplasia (NCCAH), adrenal insufficiency but not CAH (AI), and controls (C). (A) Morning (8:00-11:00 Am) concentrations of total cortisol. (B) Morning concentrations of free cortisol. (C) Total 21DF concentrations. (D) Free 21DF concentrations. n.s. nonsignificant, *P ≤ .05, **P ≤ .01, ***P ≤ .001. ^†^Excluded from statistical analysis.

In the morning samples (T0), the median total cortisol concentration in patients with untreated classic CAH was 106 nmol/L, significantly lower than patients with NCCAH (249 nmol/L, *P* = .010) and controls (202 nmol/L, *P* = .016) (Fig. 1A).

Interestingly, the median free morning cortisol concentration in patients with untreated classic CAH (8.62 nmol/L) was not significantly different from patients with NCCAH (9.84 nmol/L, *P* = .313) and controls (6.94 nmol/L, *P* = .573) (Fig. 1B). So, in contrast to the total cortisol concentrations, this suggested a comparable cortisol availability.

Patients with untreated classic CAH had a higher percentage of free cortisol (7.5%) than patients with NCCAH (5.5%, *P* = .016), patients with AI (2.1%), and controls (3.3%, *P* < .001) (Fig. S2A (23)). This indicated that patients with classic CAH had a higher free cortisol concentration than expected based on their total cortisol concentration.

### Increased 21DF Concentration in 21OHD Patients, but not in AI Patients and Controls

As expected, patients with 21OHD had higher total 21DF concentrations at T0 (median 67.5 nmol/L) than patients with NCCAH (3.03 nmol/L, *P* = .013), patients with AI (<0.70 nmol/L), and controls (<0.70 nmol/L, *P* < .001) (Fig. 1C).

Similarly, free 21DF concentrations at T0 were higher in patients with 21OHD (median 5.32 nmol/L) than in patients with NCCAH (median 0.34 nmol/L; *P* = .015), patients with AI (<0.19 nmol/L), and controls (<0.19 nmol/L, *P* < .001) (Fig. 1D). In all patients with 11OHD, both total and free 21DF concentrations were less than the LLOQ (data not shown).

### Patients With Classic CAH Had Lower (Free) Cortisol Concentrations at T60 Than Controls

**Figure 2.**
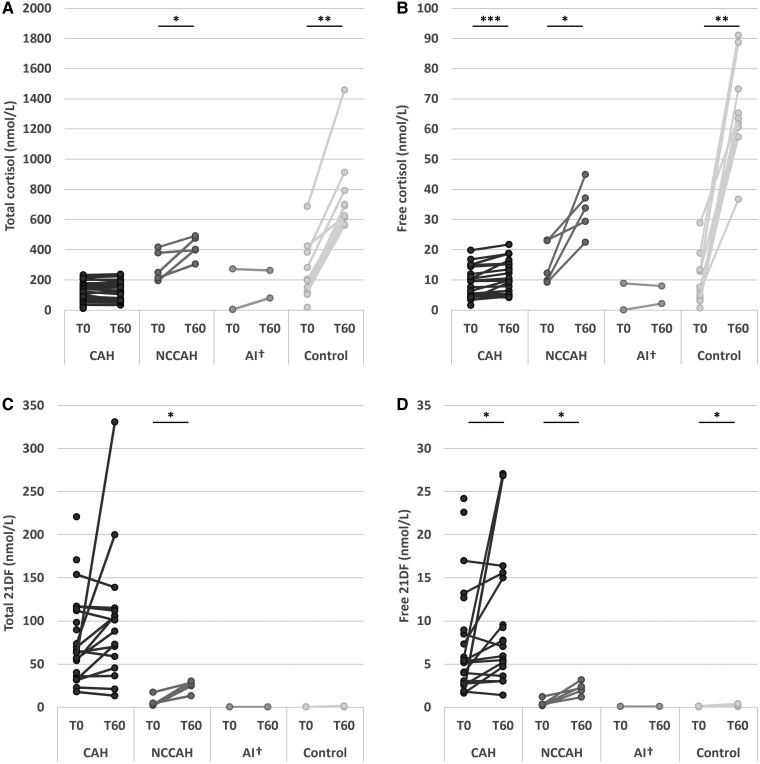
Increase in total and free cortisol and 21DF concentrations (nmol/L) before (T0) and after (T60) Synacthen in patients with classic congenital adrenal hyperplasia (CAH), nonclassic congenital adrenal hyperplasia (NCCAH), adrenal insufficiency but not CAH (AI), and controls. (A) Change in total cortisol concentrations. (B) Change in free cortisol concentrations. (C) Change in total 21DF concentrations. (D) Change in free 21DF concentrations. *P ≤ .05, ** *P* ≤ .01, ***P ≤ .001. ^†^Excluded from statistical analysis.

At T60, all controls had a total cortisol concentration ≥500 nmol/L (median 658 nmol/L), indicating sufficient cortisol production during stress according to current guidelines (5). Patients with NCCAH showed a median total cortisol increase of 42% (*P* = .043), though not as steep as in controls (198%; *P* = .005). Three out of 5 patients with NCCAH (60%) reached the 400 nmol/L threshold from the CAH guideline (13) (median 402 nmol/L). The remaining 2 patients with NCCAH had stimulated concentrations of 305 and 397 nmol/L. Total cortisol concentrations did not increase upon Synacthen administration in classic CAH (*P* = .466), nor in patients with AI (Fig. 2A). Classic CAH (median 119 nmol/L) and patients with AI (median 172 nmol/L) did both not reach the 400 or 500 nmol/L threshold (5, 13), indicating adrenal insufficiency, as expected in these patients.

The free cortisol concentration increased by 10.7% in patients with classic CAH after Synacthen administration (*P* = .001). Patients with NCCAH and controls showed a median increase of 144% (*P* = .043) and 610% (*P* = .005), respectively. No increase was observed in patients with AI (Fig. 2B). At T60, the median free cortisol concentration in patients with classic CAH was 9.85 nmol/L, which was significantly lower than in patients with NCCAH (33.8 nmol/L, *P* = .009) and controls (63.6 nmol/L, *P* < .001). Patients with AI had a median of 5.08 nmol/L (data not shown).

All controls had free cortisol increases ≥37 nmol/L after stimulation. Patients with NCCAH had free cortisol concentrations ranging from 22 to 45 nmol/L after Synacthen stimulation. Patients with classic CAH did not reach free cortisol concentrations seen in controls (maximum 21.8 nmol/L).

After Synacthen, the percentage of free cortisol increased in classic CAH (*P* = .001), NCCAH (*P* = .043), and control patients (*P* = .005), but not in patients with AI (Fig. S2B (23)). At T60, the median free cortisol percentage in patients with classic CAH (8.9%) was not significantly different from patients with NCCAH (7.4%, *P* = .211) and controls (9.7%, *P* = .499). The median free cortisol percentage in patients with AI was 2.9% (Fig. S2C (23)).

### Concentrations of Free 21DF Increased After Synacthen Administration in Patients With Classic 21OHD

After Synacthen administration, total 21DF concentrations significantly increased in patients with NCCAH (median at T60 26.4 nmol/L, *P* = .043), but not in patients with classic 21OHD (median at T60 94.6 nmol/L, *P* = .121) (Fig. 2C). In patients with AI, total 21DF concentrations remained below the LLOQ at T60 and in controls, the maximum was twice the LLOQ (0.7 nmol/L) for 2 patients. Patients with classic 21OHD had significantly higher total 21DF concentrations at T60 than controls (*P* < .001) (data not shown).

Free 21DF concentrations significantly increased in both classic CAH (*P* = .023) and NCCAH patients (*P* = .043) (Fig. 2D). At T60, the median free 21DF concentration in patients with classic CAH was 7.34 nmol/L, which was significantly higher than in patients with NCCAH (median 2.22 nmol/L, *P* = .041). In patients with AI and controls, median free 21DF concentrations were below the LLOQ at T60 (data not shown). In all patients with 11OHD, both total and free 21DF concentrations were below the LLOQ (data not shown).

In patients with classic 21OHD, the percentage of free 21DF increased from a median of 8.4% at T0 to 10.3% at T60 (*P* = .035). There was no change in the median free 21DF percentage in patients with NCCAH (*P* = .893), remaining at 7.9% both at T0 and T60 (data not shown).

## Discussion

This is the first study that evaluated free cortisol and free 21DF concentrations in patients with untreated classic CAH (both 21OHD and 11OHD), NCCAH, other forms of AI, and controls. We found that despite lower total cortisol concentrations, free cortisol concentrations were similar in untreated patients with classic CAH and controls in the unstimulated state. This could explain the mild or absent symptoms of cortisol deficiency in some patients with classic 21OHD or 11OHD. In addition, patients with classic 21OHD had elevated concentrations of total and free 21DF, which is not observed in patients with AI and controls, increasing the glucocorticoid activity. After Synacthen, patients with classic CAH showed no increase in total cortisol concentrations and minimal increase in free cortisol concentrations, but patients with classic 21OHD had increased free 21DF concentrations. This might explain why the described patients survived periods of stress without glucocorticoid treatment.

We developed an LC-MS/MS method to measure free cortisol and free 21DF. The mean free cortisol percentage in controls (3.7%) was consistent with previous findings (3.95%) (31, 32). The relative increase in free cortisol concentration (610%) after Synacthen was greater than the relative increase in total cortisol (198%), as observed in other studies (31-33). Therefore, evaluating free concentrations may provide more informative insights than total concentrations.

Glucocorticoids perform numerous functions, and they are particularly necessary during stress. Thus, glucocorticoid levels should be interpreted in 2 contexts: the daily baseline state and the state under physical stress.

Cortisol in blood is mostly bound to CBG and albumin, but only its free form is biologically active (34). In the baseline state, we found that while total cortisol concentrations were lower in untreated patients with classic CAH than in controls, free cortisol concentrations were similar. This is supported by data from Waaijers et al, who found cortisol concentrations within the reference range in hair samples from untreated patients with classic CAH (35), indicating comparable long-term free cortisol levels. Possible explanations for this are (1) increased levels of structurally analogous steroids, like 17OHP and 21DF, may disrupt or prevent cortisol from binding to CBG or albumin, leading to a higher percentage of free cortisol (15, 21, 22); (2) increased concentrations of adrenal androgens in patients with CAH may decrease 11β-hydroxy steroid dehydrogenase type 2 activity, preventing cortisol conversion to inactive cortisone (36); (3) androgens may lower CBG levels, potentially increasing the free cortisol concentrations (37, 38).

In addition, patients with classic 21OHD had increased 21DF concentrations in the basal state, confirming previous findings (39-41). Our group showed that 21DF has 49% of cortisol's potency in activating the glucocorticoid receptor (17). The median free 21DF concentration in these patients (5.32 nmol/L) is hypothetically equivalent to (0.49 × 5.32 =) 2.61 nmol/L free cortisol, potentially increasing glucocorticoid activity beyond what total cortisol concentrations suggest. This could explain why some patients with classic CAH have managed without glucocorticoid medication during daily circumstances. Patients with 11OHD are not capable of producing 21DF, but their free cortisol concentrations were already similar to controls.

During physical stress, such as illness or surgery, the demand of glucocorticoid increases. Patients with classic CAH were not able to increase their total cortisol concentrations after ACTH stimulation. In our study, these patients did not reach the 400 to 500 nmol/L total cortisol threshold after Synacthen administration (13). They also showed only a minor increase in free cortisol concentrations, which did not match the increase observed in controls. Interestingly, patients with 21OHD exhibited a significant rise in free 21DF up to a median of 7.34 nmol/L. Given its ability to activate the glucocorticoid receptor, this increase may significantly enhance glucocorticoid activity. However, whether this concentration of free glucocorticoids is sufficient to prevent an Addisonian crisis remains uncertain. This raises questions about how previously described patients with untreated classic 21OHD have managed physical stress, such as dengue fever and surgery, without glucocorticoid treatment (17-20). Other protective measures might contribute to their resilience during stress. For instance, androgens may enhance glucocorticoid activity either directly or by facilitating glucocorticoid receptor binding (36). Additionally, synthetic ACTH administration has been shown to increase free cortisol fractions in healthy individuals (34). The continuous ACTH pressure in patients with classic CAH might also boost free cortisol fractions and glucocorticoid activity. Further research is needed to explore these possibilities in patients with CAH.

Some patients with classic CAH have survived without glucocorticoid treatment despite low total cortisol concentrations and inability to increase cortisol during stress (17-20). This observation may be explained by the similar free cortisol concentrations in patients with untreated classic CAH and in controls. In clinical practice, some patients with classic CAH, particularly 46,XY males and 46,XX individuals living as males, show minimal symptoms of adrenal insufficiency and may refuse glucocorticoid therapy (17, 42). However, untreated patients face significant risks, including an Addisonian crisis, development of testicular adrenal rest tumors, formation of adrenal myelolipomas, and adverse cardiovascular effects (43, 44).

Genotype–phenotype correlations in CAH are not always accurate. Our study also revealed discrepancies between genetic predictions and clinical phenotype, especially with the R356W mutation in *CYP21A2*. Stoupa et al (45) reported that 60% of their patients with NCCAH had an inadequate cortisol response to Synacthen, while none of them experienced periods of cortisol deficiency, highlighting that insufficient total cortisol concentrations do not always correlate with clinically relevant adrenal insufficiency.

Our findings suggest that measuring total cortisol concentrations alone may not adequately assess adrenal cortex function in all forms of PAI. Additional measurement of free cortisol may provide a more accurate reflection of glucocorticoid activity. Furthermore, in patients with 21OHD, evaluating (free) 21DF concentrations should be considered to comprehensively evaluate the glucocorticoid activity. This approach is particularly useful for patients with milder forms of CAH to indicate the necessity of glucocorticoid treatment. More research is necessary to compute cutoff values for defining adrenal cortex insufficiency and to integrate these measures into clinical practice. Measuring free cortisol concentrations is also relevant for other conditions where discrepancies between free and total cortisol are present, such as hypoproteinemia in critical illness (34), CBG deficiency (14), and oral contraceptive use (46).

Our study has several strengths and limitations. It is the first to quantify free cortisol and free 21DF concentrations in patients with untreated classic CAH and compare these levels with controls, patients with NCCAH, and patients with AI. We used a state of the art dialysis method to obtain free concentrations followed by a sensitive LC-MS/MS method, which is the gold standard for serum steroid measurements (13). Furthermore, deuterium or ^13^C-isotopes of each steroid were used as internal standards to allow for the correction of matrix effects. Moreover, all included patients with CAH were genetically confirmed. Limitations include the low number of patients, especially for patients with AI. Therefore, this group was excluded from statistical analyses. Cortisol has a diurnal rhythm, so we included only samples taken before 11.00 Am for the analysis of cortisol concentrations to mitigate this issue. The control group had a higher percentage of males than the patient groups. In clinical practice, there is no sex separation for cutoff values (5, 13), because there are no clinically relevant differences in concentrations of cortisol and 21DF between males and females (47, 48). Therefore, we did not stratify for sex.

To conclude, patients with untreated classic CAH have basal state free cortisol concentrations in the same range as controls. Moreover, patients with 21OHD produce elevated concentrations of total and free 21DF, a precursor steroid with glucocorticoid activity. Both mechanisms might explain their relatively mild symptoms of adrenal insufficiency. During stress, patients with 21OHD exhibit increased (free) 21DF concentrations, offering potential additional protection compared with other forms of PAI who did not produce 21DF. Future research should focus on the role of free cortisol and free precursor steroids, especially in milder forms of CAH to better assess the glucocorticoid activity and determine the need for glucocorticoid treatment.

## Data Availability

Some or all datasets generated during and/or analyzed during the current study are not publicly available but are available from the corresponding author on reasonable request.

## Abbreviations

11OHD: 11-hydroxylase deficiency
17OHP: 17-hydroxyprogesterone
21DF: 21-deoxycortisol
21OHD: 21-hydroxylase deficiency
ACTH: adrenocorticotropic hormone
AI: adrenal insufficiency
CAH: congenital adrenal hyperplasia
CBG: corticosteroid-binding globulin
LC-MS/MS: liquid chromatography tandem mass spectrometry
LLOQ: lower limit of quantification
NCCAH: nonclassic CAH
PAI: primary adrenal insufficiency
SPE: solid phase extraction
